# Antilipoxygenase and Anti-Inflammatory Activities of *Streblus asper* Leaf Extract on Xylene-Induced Ear Edema in Mice

**DOI:** 10.1155/2020/3176391

**Published:** 2020-12-05

**Authors:** Kanathip Singsai, Pimchanok Charoongchit, Waritsara Chaikaew, Nirut Boonma, Pitsinee Fhanjaksai, Karitta Chaisatan

**Affiliations:** ^1^School of Pharmaceutical Sciences, University of Phayao, Phayao 56000, Thailand; ^2^Unit of Excellence of Pharmacological Research and Vaccine Development in Animal Models, University of Phayao, Phayao 56000, Thailand; ^3^Chiangrai Prachanukroh Hospital, Chiangrai 57000, Thailand; ^4^Paolo Hospital Phaholyothin, Bangkok 10400, Thailand; ^5^Uttaradit Hospital, Uttaradit 53000, Thailand; ^6^Lampang Hospital, Lampang 52000, Thailand

## Abstract

*Streblus asper* (SA) belonging to the Moraceae family is well known as a folk medicinal plant in Asian countries. This study aimed to investigate the antilipoxygenase activity and the anti-inflammatory effects of the SA leaf extract. An in vitro antilipoxygenase study was performed using a lipoxygenase assay, and the oxidation of linoleic acid into 13-hydroperoxy linoleic acid (HPODE) was detected with a UV spectrophotometer at a wavelength of 234 nm. In the animal study, twenty-five male ICR mice were induced as ear edema by topical xylene, and the ear thickness of the mice was measured. The lipoxygenase assay results showed that the IC50 values of diclofenac sodium and SA were 0.0015 and 37.96 *μ*g/mL, respectively. In the animal study, mice that received diclofenac sodium exhibited significantly reduced ear edema induced by xylene from 30 min onward, while mice that received 250 mg/kg and 500 mg/kg SA exhibited significantly reduced ear edema compared with the control group 45 min after induction with xylene. These results suggested that the SA leaf extract had anti-inflammatory activity. However, further studies are required to evaluate these effects and the additional potential of the plant that might be beneficial for the development of pharmaceutical products that prevent and treat inflammation.

## 1. Introduction

The inflammation process is a defense mechanism of living tissues triggered by trauma, pathogens, stress, toxic substances, and cell damage. It is a complex reaction involved in resolving the stimuli and initiating the healing process [1]. If acute inflammation is uncontrolled, chronic inflammation might be progressive and contribute to chronic inflammatory diseases [1, 2]. Nonsteroidal anti-inflammatory drugs (NSAIDs), steroids, and opioids are accepted and widely used for the management of inflammatory symptoms or to treat diseases associated with inflammation [3]. However, they are still used with awareness and under consideration by physicians due to their side effects. Within the past century, plants and their extracts have gained much attention as a new source for alternative anti-inflammatory therapeutic purposes [4, 5]. Their pharmacological activities and phytochemical constituents have been increasingly investigated.

*Streblus asper* (SA), belonging to the Moraceae family, is well known as a folk medicinal plant in Asian countries, such as India, Sri Lanka, Malaysia, the Philippines, Southern China, and Thailand. It is a rich source of cardiac glycosides, phenolic compounds, and volatile oils [6]. Several scientific studies have reported its pharmacological activities, including antibacterial, antiseptic, antidiarrheal, antidiabetic, antioxidant, and anti-Parkinson's activities [6–8]. In addition, a SA leaf extract demonstrated an inhibitory effect on carrageenan-induced paw edema in rats. The possible mechanism of this effect was related to the suppression of the LPS-induced expression of COX-2 and iNOS mRNA [9]. Scientific evidence on the anti-inflammatory effects of SA extracts has rarely been reported, and there is no confirmed evidence of an anti-inflammatory mechanism through the lipoxygenase enzyme. Therefore, the purpose of this study was to evaluate the anti-inflammatory activity of an aqueous SA leaf extract, including in vitro lipoxygenase assay and in vivo experiments on xylene-induced ear edema in mice. The results of this study will provide potential information for the treatment of neurogenerative disorders caused by neuroinflammation [10].

## 2. Materials and Methods

### 2.1. Animals

In this study, adult male ICR mice (40–60 g) were used. The mice were housed in a room maintained at 25 ± 2°C on a 12 h light/dark cycle. All experiments were carried out according to the guidelines of the Institute of Animals for Scientific Purposes Development Chemicals (IAD). Animal ethics no. 620104001 was approved by the Lab Animal Research Center, University of Phayao.

### 2.2. Preparation of the Extract

The fresh SA leaves were collected from the botanical garden of the School of Pharmaceutical Sciences, University of Phayao (Phayao, Thailand). The fresh leaves were desiccated, crushed, and weighed. For aqueous extract preparation, the dried mashed powder was soaked in deionized water at 60°C for 6 hours, filtered, and lyophilized. The percentage yield of the SA extract was 14.59% of the dried leaves. The dry powdered extract was kept in airtight, light-protected containers at 2–4°C and dissolved in distilled water before being used [7].

### 2.3. Phytochemical Screening

Phytochemical screening of the SA extract followed the method of Farnworth [11] and afforded flavonoids, triterpenoids, cardiac glycosides, and saponins. Flavonoids and triterpenoids were detected using Shinoda's test and the Liebermann–Burchard test. Cardiac glycosides, including the steroid nucleus, unsaturated lactone ring, and 2-deoxy sugar, were detected using the Liebermann–Burchard test, Kedde's reagent, and Keller–Kiliani test, respectively. The forth test was used to detect saponins.

### 2.4. Antilipoxygenase Activity of SA

An in vitro anti-inflammatory study was performed using a lipoxygenase assay adapted from Leelaprakash et al. and Chung et al. [12, 13]. In brief, the oxidation of linoleic acid into 13-hydroperoxy linoleic acid (HPODE) was detected by UV spectrophotometry at a wavelength of 234 nm. Diclofenac sodium as a standard was prepared at concentrations of 0.001, 0.01, 0.1, and 1 *μ*g/mL, and the SA extract was prepared at concentrations of 5, 10, 25, 50, and 100 *μ*g/mL. The % inhibition was calculated and expressed as the mean ± SEM of three replicates.

### 2.5. Anti-Inflammatory Effects of SA on Xylene-Induced Ear Edema

The study of the anti-inflammatory effects of SA on xylene-induced ear edema followed the experimental method of Sadeghi et al. and Anyasor and Ijituyi [14, 15]. Twenty-five male ICR mice were randomly divided into five groups. Group 1 (control group) mice were fed distilled water. Group 2 (positive control group) mice were fed 10 mg/kg diclofenac sodium. The mice in groups 3–5 were fed 125 mg/kg, 250 mg/kg, or 500 mg/kg SA extract, respectively. All mice were fed once daily for 7 days. On day 8, inflammation was induced in the animals as ear edema by topical xylene, and the mice were fed distilled water, diclofenac sodium, or SA extract 15 min later, after which the mice were induced by xylene. The right ear thickness of the mice was measured with a digital thickness gauge meter after 15, 30, 45, and 60 min.

### 2.6. Statistical Analysis

Statistical analysis was performed using SigmaPlot (version 14.0). The data were analysed by one-way analysis of variance (ANOVA) followed by Tukey's multiple comparisons test. The criterion for statistical significance was set at *p* < 0.05.

## 3. Results and Discussion

### 3.1. Phytochemical Analysis

Phytochemical screening of the SA extract afforded flavonoids, triterpenoids, cardiac glycosides, and saponins (Table 1).

Phytochemicals in SA comprise cardiac glycosides [16], flavonoids, triterpenoids, and saponins, which might be responsible for the distinct anti-inflammatory activities of the extract [17]. Flavonoids are useful in acute inflammation [18] and act by inhibiting arachidonic acid release, which is central in prostaglandin synthesis [19, 20]. Triterpenoids may exert their anti-inflammatory actions by decreasing iNOS expression [21, 22]. A previous study reported that *Streblus asper* (SA), as a potential anti-inflammatory agent, significantly dose-dependently inhibited paw edema and reduced the mRNA expression of cyclooxygenase (COX)-2 and inducible nitric oxide synthase (iNOS) in RAW 264.7 cells [9].

### 3.2. Effects of the SA Extract on Antilipoxygenase Activity

The lipoxygenase assay results showed that the percentage inhibition of the lipoxygenase activity by the SA extract is shown in Table 2. The IC50 values of diclofenac sodium (as a standard) and the SA extract were 0.0015 and 37.96 *μ*g/mL, respectively.

Lipoxygenase is the enzyme involved in the arachidonic acid pathway that produces leukotrienes [23]. This study was performed to investigate the antilipoxygenase activity of a SA leaf extract in vitro. The results showed that the SA extract has less antilipoxygenase activity than diclofenac sodium. It is possible that SA has anti-inflammatory activity via inhibition of cyclooxygenase as the main pathway, while lipoxygenase is involved in a minor pathway that slightly involves leukotrienes; therefore, the proposed mechanism of the anti-inflammatory action of SA might involve prostaglandins, which are products of the cyclooxygenase pathway.

Many studies reported that phenolic compounds inhibit the inflammatory process via inhibiting lipoxygenase enzyme involved in transformation of arachidonic acid to inflammatory mediators and involved free radical scavenging in arachidonic acid metabolism [18, 24]. In addition, flavonoids have antioxidative activity by decreasing capillary permeability, disturbing arachidonic acid pathway, and inhibiting cyclooxygenase and lipoxygenase enzymes resulting in a decreased level of prostaglandin and leukotriene [19]. From our previous study, the certain polyphenolic compounds such as gallic acid, isoquercetin, quercetin, rutin, catechin, and tannic acid were found in SA aqueous extracts. Therefore, this can be explained that the antilipoxygenase activity of the SA extract might be affected by the antioxidative action of phenolic compounds [25].

### 3.3. Effects of the SA Extract on Xylene-Induced Ear Edema in Mice

The mouse ear thickness values (expressed as the mean ± SE) are shown in Table 3. The thickness of the ears was used to calculate the percentage edema from the following equation.

(1) $$\begin{array}{c} \%\text{edema}=\frac{\left(T_{t}-T_{0}\right)}{T_{0}}\;\times 100, \end{array}$$

where *T*_*t*_ represents the ear thickness at *t* min, and *T*_0_ represents the ear thickness at 0 min.

**Figure 1:**
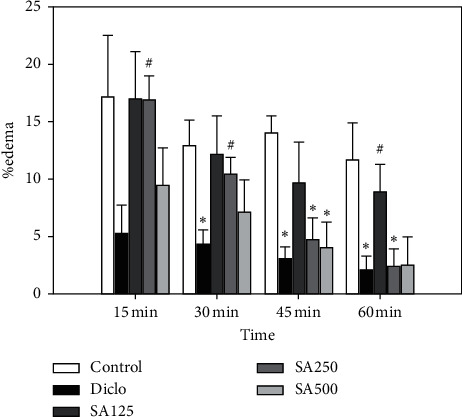
Percentage ear edema induced by xylene at 15, 30, 45, and 60 min.

The results showed that the administration of diclofenac sodium (10 mg/kg) significantly reduced ear edema induced by xylene from 30 min onward, while the mice that received 250 mg/kg and 500 mg/kg SA significantly reduced ear edema compared with the control group 45 min after induction by xylene (Figure 1).

The figure represented that the administration of diclofenac sodium (10 mg/kg) significantly reduced ear edema induced by xylene from 30 min onward, while the mice that received 250 mg/kg and 500 mg/kg SA significantly reduced ear edema compared with the control group 45 min after induction by xylene.

- *∗p* < 0.05, different from the control group.
- #*p* < 0.05, different from the diclofenac group.

Animal experiments and studies of the anti-inflammatory effects of SA on xylene-induced ear edema in mice indicated that SA has anti-inflammatory activity by reducing ear edema in a dose-dependent manner; however, the onset of action of SA is slower compared to diclofenac sodium. This study represents the acute anti-inflammatory effect of the SA extract in which innate immune cells form the first line of immune defense and regulate activation of adaptive immune responses. Most of the features of acute inflammation continue as the inflammation becomes chronic, including the expansion of blood vessels (vasodilation), increase in blood flow, capillary permeability, and migration of neutrophils into the infected tissue through the capillary wall (diapedesis) [26]. Xylene-induced edema partially involved substance *P* as a common inflammatory model for increasing capillary permeability and leukocyte infiltration [27]; thus, the proposed mechanism of SA might reduce the release of substance *P* or antagonize its action in the inflammatory process. During the initial phase, also called neurogenic phase, substance *P* and bradykinin are released. Substance *P*, a neurotransmitter in the central nervous system, induced nitric oxide releasing be the cause of vasodilation and plasma exudation [14, 15]. Therefore, anti-inflammatory activity of SA might be referring to neurogenic inflammation.

## 4. Conclusions

The present study indicated that the SA leaf extract had anti-inflammatory activities, including antilipoxygenase activity and reduced mouse ear edema. However, further studies are required to evaluate the chronic inflammatory activities and the additional potential of the plant that might be beneficial for the development of pharmaceutical products that prevent and treat inflammation.

## Figures and Tables

**Table 1:** Phytochemical screening of the SA extract.

Phytochemical screening	Test	Results
Flavonoids	Shinoda's test	Yes
Triterpenoids	Liebermann–Burchard test	Yes

Cardiac glycosides		
Steroid nucleus	Liebermann–Burchard test	Yes (triterpenoid)
Unsaturated lactone ring	Kedde's reagent	Yes
2-Deoxy sugar	Keller–Kiliani test	No

Saponins	Forth test	Yes

**Table 2:** Percent inhibition of lipoxygenase activity by the SA extract.

SA extract concentration (*μ*g/mL)	%inhibition (mean ± SEM)
5	17.16 ± 0.50
10	24.62 ± 1.73
25	34.38 ± 5.64
50	81.04 ± 0.74
100	92.96 ± 7.08

**Table 3:** Ear thickness (mm) express as mean ± SE.

Group	Ear thickness (mm)				
	Baseline	15 min	30 min	45 min	60 min
Control	0.238 ± 0.005	0.278 ± 0.009	0.263 ± 0.003	0.273 ± 0.003	0.265 ± 0.006
Diclofenac	0.248 ± 0.005	0.258 ± 0.002	0.250 ± 0.004	0.250 ± 0.004*∗*	0.248 ± 0.004
SA 125	0.250 ± 0.003	0.292 ± 0.007#	0.280 ± 0.006#	0.274 ± 0.007#	0.272 ± 0.004#
SA 250	0.260 ± 0.004*∗*	0.304 ± 0.007*∗*, #	0.290 ± 0.009#	0.272 ± 0.004#	0.266 ± 0.004#
SA 500	0.260 ± 0.005*∗*	0.284 ± 0.002#	0.278 ± 0.004#	0.270 ± 0.003#	0.266 ± 0.002#

The control group was administered distilled water. Statistical differences from the control and diclofenac groups were determined by ANOVA followed by Tukey's test. *∗p* < 0.05, different from the control group. #*p* < 0.05, different from the diclofenac group.

## Data Availability

The data used to support the findings of this study are available from the corresponding author upon request.
